# The Effect of Bone Morphogenetic Protein 4 on the Differentiation of Mouse Embryonic Stem Cell to Erythroid Lineage in Serum Free and Serum Supplemented Media

**Published:** 2009-09

**Authors:** Mohammad Ali Owchi, Mojdeh Salehnia, Mehdi Forouzandeh Moghadam, Mandana Beigi Boroujeni, Ebrahim Hajizadeh

**Affiliations:** 1 *Department of Anatomy, Tarbiat Modares University, Iran*; 2 *Department of Biotechnology, Tarbiat Modares University, Iran*; 3 *Department of Anatomy, Faculty of Medical Sciences, Lorestan University of Medical Sciences, Khoramabad, Iran*; 4 *Department of Biostatistic, Tarbiat Modares University, Iran*

**Keywords:** embryonic stem cells, bone morphogenetic protein-4, erythropoiesis, serum free media

## Abstract

This study was done to compare the effects of bone morphogenetic protein-4 (BMP-4) on mouse embryonic stem cells (ESC) differentiation to erythroid lineage in serum free and serum supplemented media. The embryoid bodies (EBs) cells were seeded in semisolid serum free and serum supplemented media in the presence of different concentrations of BMP-4. The erythroid colonies were assessed morphologically, ultrastructurally and by benzidine staining. The expression of the epsilon (ε), βH1 and βmajor globins, Runx1 and β2m genes was evaluated by Real time PCR. The colony size and the percent of benzidine-positive colonies increased in both BMP-4 supplementd groups but the number of colonies were lower in these groups than control. Erythropoiesis related genes were expressed in both serum free and serum supplemented groups. There were not significant differences between the ratios of genes expression to β2m in these groups except the ratio of Runx1 was significantly higher in serum free group (*P*<0.05). The ratio of ε and βH1 to β2m in EBs was higher than both BMP-4 containing groups (*P*<0.05) and βmajor was not expressed in EB cells. These findings showed in serum free condition the effects of BMP-4 on the erythroid differentiation was prominent than serum supplemented group.

## INTRODUCTION

Embryonic stem cells (ESC) have the potential to produce all cell types of the body and they could be a valuable model to study the molecular events during cell growth and differentiation *in vitro* (1–3). There are several reports on the differentiation induction of variety of ESC lines to hematopoietic and erythroid cell lineage (4–9).

Differentiation of ESC into hematopoietic cell types depends upon the addition of some cytokines and growth factors to semisolid culture media which allowed the development of embryoid bodies derived cells in either defined serum free media or serum supplemented media (10–14). These effective factors are including erythropoietin, bone morphogenetic proteins 4 (BMP-4), stem cells factor (SCF) and vascular endothelial growth factor (VEGF). They have been used in combination with each others or in co-culture with different lines of stromal cells (15–18).

BMP-4 is a multi functional growth factors that belong to the transforming growth factor beta superfamily. BMP-4 binds to a family of heteromeric cell-surface receptors consisting of type I and type II serine/threonine protein kinase subunits (19). BMP-4 is a key growth factor for maintenance of hematopoietic stem cells (HSC) and its role in hematopoiesis has also been demonstrated using mouse ES cell *in vitro* differentiation systems (5, 9–12, 20, 21). It has been shown that BMP-4 is essential for the generation of multiple hematopoietic lineages including erythroid, myeloid, and lymphoid cells from ES cells in chemically defined or serum free media (22, 23).

Li *et al*. (20) found that BMP-4 was an important factor in the induction of the hematopoietic differentiation of rhesus monkey ES cells in the culture condition which supplemented with horse serum and fetal bovine serum (FBS). Under this condition the number of primary hematopoietic clusters increased by an average of 15 fold. Also they indicated that BMP-4 treated ES cells demonstrated an up-regulation of genes responsible for embryonic hematopoiesis and angiogenesis during 7 days of culture (20).

Pick *et al*. (11) evaluated the roles of four growth factors, BMP-4, VEGF, SCF and basic fibroblast growth factor (FGF) on the production of hematopoietic cells from human ESC. They found that BMP-4 initiated the expression of related hematopoietic genes and supported the generation of hematopoietic progenitor cells at a low frequency, and the combination of BMP-4, VEGF, SCF and FGF further enhanced the yield of hematopoietic cells (11).

Chadwick *et al*. (12) reported the treatment of human ESC during embryoid bodies (EBs) formation with cytokines and BMP-4 strongly promotes hematopoietic differentiation. Addition of BMP-4 had no statistically significant effect on hematopoietic differentiation but enabled significant enhancement in progenitor self-renewal, independent of cytokine treatment.

Most of the mentioned studies have been done to understand the effects of BMP-4 in hematopoietic differentiation of ESC in different culture conditions such as in serum supplemented or defined serum free media in combination with other growth factors (5, 10–12).

However there was little attention to its effects on the erythroid differentiation without other kinds of growth factors. Thus the aim of this study was to evaluate and compare the effects of different dosage of BMP-4 on mouse ESC induction to the erythroid lineage in serum free and serum (fetal bovine serum) supplemented media. Also we tried to determine the erythropoiesis pattern (primitive and definitive) in differentiated cells by analysis of the specific erythropoiesis related gene expression using real time PCR technique.

## MATERIALS AND METHODS

### Cell culture and embryoid body formation

CCE ESCs were cultured in Dulbecco's modified Eagle's medium (DMEM; Gibco, UK) with 20% fetal bovine serum (FBS; Gibco, UK), 0.1 mM non-essential amino acids (Gibco, UK), 0.1 mM β-mercaptoethanol (Sigma, USA) and 1000 U/ml leukemia inhibitory factor (LIF; Sigma, USA). Undifferentiated ESCs were passaged every two days by trypsin (0.25%; Merck, Germany)/EDTA (1 mM; Sigma, USA) dissociation and cultured at 37°C, 5% CO_2_, and 95% humidity (15, 24).

Two days prior to their differentiation, ESCs were cultured in Iscove's modified Dulbecco's medium (IMDM; Sigma, USA) with FBS (15%) and monothioglycerol (MTG) (1.5 × 10^−4^ mol/L; Sigma, USA), ascorbic acid (50 ng/mL; Sigma, USA), and l-glutamine (2 mmol/L; Gibco, UK). After reaching confluence, the cells were dissociated with trypsin/EDTA and plated on plastic culture dishes for 5 days in the absence of LIF to promote EBs formation (5 × 10^3^ cells/ml) (24,25).

### Experimental design

After dissociation of EBs cells with trypsin/EDTA, their erythroid differentiation was compared in the following experimental and control groups: A; in semisolid serum free media containning different concentrations of BMP-4 (5, 10, 20 and 40 ng/ml) and B; in semisolid media supplemented with FBS and different concentrations of BMP-4 (5, 10, 20 and 40 ng/ml). The media without BMP-4 were considered as their controls. Then the expression of the epsilon (ε), βH1 and βmajor globins, Runx1 and β2m genes was evaluated by Real time PCR in the group with high perent of erythroid like colonies.

### Differentiation of EBs cells in semisolid serum free media containing different concentrations of BMP-4

One milliliter of semisolid serum free medium (methylcellulose 1%; Sigma, USA) contained 5 × 10^4^ cells in IMDM, knock out serum replacement (30%; Gibco, UK), MTG (4.5 × 10^−4^ mol/L; Sigma, USA), ascorbic acid (12.5 ng/ml), l-glutamine (2 mmol/L; Sigma, USA), penicillin (100 U/ml; Gibco, UK), streptomycin (100 µg/ml; Gibco, UK), and different concentration of BMP-4 (0, 5, 10, 20 and 40 ng/ml; R&D system, USA).

### Differentiation of EBs cells in semisolid media supplemented with serum and different concentrations of BMP-4

One milliliter of semisolid medium (methylcellulose 1%; Sigma, USA) contained 5 × 10^4^ cells in IMDM, FBS (30%; Gibco, UK), MTG (4.5 × 10^−4^ mol/L; Sigma, USA), ascorbic acid (12.5 ng/ml), l-glutamine (2 mmol/L; Sigma, USA), penicillin (100 U/ml; Gibco, UK), streptomycin (100 µg/ml; Gibco, UK), and different concentration of BMP-4 (0, 5, 10, 20 and 40 ng/ml; R&D system, USA).

### Colony forming assay and benzidine staining

Colony assays were carried out after 14 days for all experimental and control groups. The size and total number of colonies were evaluated using an inverted microscope. The number and percent of erythroid colonies determined morphologically and using benzidine staining.

Benzidine dihydrochloride (Sigma, USA) staining to identify hemoglobin-containing cells was carried out according to the Palis method (24), with slight modification. Briefly, 20 µl of 30% hydrogen peroxide was added to 1 ml of benzidine immediately before use. A 200µl aliquot of the resulting solution was added to the colonies, which were then examined using an inverted microscope. Benzidine-positive colonies stained dark blue in result of the reaction between hemoglobin and H_2_O_2_, while benzidine-negative colonies were light yellow (26).

### Electron microscopy

The group with high percent of erythroid colonies were selected for transmission electron microscopy (TEM) evaluation therefore, according to the statistical analysis the colonies were collected from semisolid serum free media group containing 20ng/ml of BMP-4 and fixed in 2.5% glutaraldehyde in PBS (pH 7.4) for 2 h and post-fixed with 1% osmium tetroxide in the same buffer for 2 h. After dehydration in a graded ethanol series, they were placed in propylene oxide and embedded in Epon 812. Semi-thin sections (0.5 μm) were stained with toluidine blue for light microscopy. Ultrathin sections were stained with uranyl acetate and lead citrate and examined by transmission electron microscopy (Zeiss EM 900).

### Real time polymerase chain reaction (Real time-PCR)

After collection of the data and statistical analysis of all experimental and control groups at the first step of study, the colonies formed in 20 ng/ml of BMP-4 in serum free and serum supplemented media were pooled on day 5 of culturing and evaluated for epsilon (ε), beta H1 (βH1) and βmajor globin, Runx1 and β2m genes expression. Also the undifferentiated embryoid bodies were considered as control.

Total RNA was extracted using Qiagen kit (USA) according to the manufacturer's instructions and the extracted RNA integrity was confirmed using gel electrophoresis and concentration of each sample was determined by ultraviolet spectrophotometry. After RNA extraction, DNase treatment was done immediately to eliminate genomic DNA contamination according to Fermentas RNase free-DNase kit. Total RNA was stored at −80°C and triplicates were prepared for each colony sample.

After extraction of total RNA, one step real time PCR was performed on Rotor-gene 3000 (Corbett) real time thermal Cycler according to QuantiTect SYBR Green RT-PCR kit (Qiagen; Catalog no.204243).

For target sequence amplifications, 500ng of RNA was used per 25 μL reaction volume. After completing the PCR run, melt curve analysis was used to confirm the amplified product. The real time PCR products were further verified by agarose gel electrophoresis. For each sample, the reference gene (β2M) and the target genes (ε, βH1, Runx1 and βmajor) were amplified in the same run. Standard curves were obtained by using the logarithmic dilution series of total RNA. Then relative quantitation of target genes was determined by Pfaffl method. The primers sequences for amplification of erythropoiesis related genes are listed in the Table 1 (15).

**Table 1:** Sequence of the primers

Gene	Sense	Antisense	Size

β2M	5′TGACCGGCTTGTATGCTATC-3′	5′CACATGTCTCGATCCCAGTAG-3′	316 bp
εglobin	5′GGAGAGTCCATTAAGAACCTAGACAA-3′	5′CTGTGAATTCATTGCCGAAGTGAC-3′	122 bp
βH1	5′AGTCCCCATGGAGTCAAAGA-3′	5′CTCAAGGAGACCTTTGCTCA-3′	265 bp
RUNX1	5′AAACAAAACTGACCCGCAAG-3′	5′CAAAGTCAAATGCCCAACAG-3′	217 bp
βMajor	5′CACCTTTGCCAGCCTCAGTG3′	5′CTCAAGGAGACCTTTGCTCA3′	200 bp

### Statistical analysis

Statistical analysis was done with SPSS software. Data are shown as mean ± SD or as indicated. Colony assays were repeated eight times and the results were compared by Tukey and Pearson Chi-Square tests. The Real time PCR experiments were repeated three times and the results were compared by LSD test (*P*<0.05).

## RESULTS

### Morphological observation

**Figure 1:**
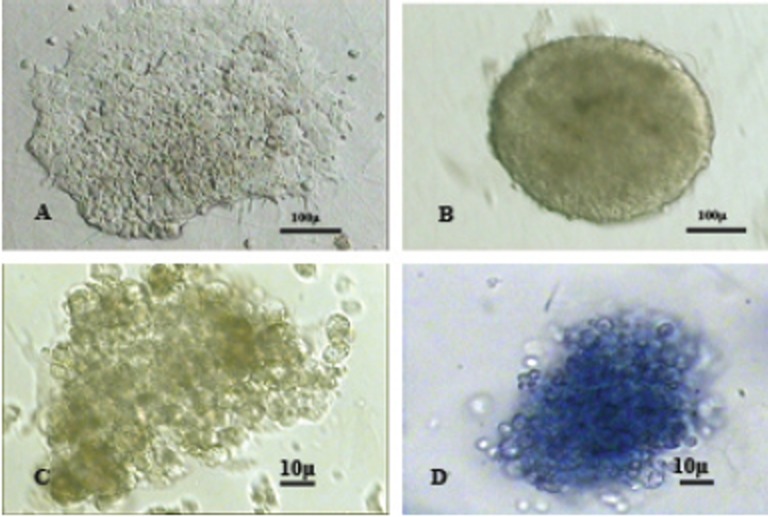
A, The undifferentiated colony of CCE embryonic stem cells; 3100 magnification; B, Four-day-old embryoid body; 3400 magnification; C, The photograph of erythroid colony; 3100 magnification; (D) Benzidine-positive colony; 3200 magnification.

Cultured CCE mouse embryonic stem cells (CCE) formed colonies with irregular borders in undifferentiated state (Fig. 1A). Day five embryonic bodies were spherical with regular borders (Fig. 1B).

The colonies with morphology similar to erythroid colonies (BFU-e) were illustrated in Fig. 1C. These colonies were benzidine positive and dark blue on the 14th day of culturing in semisolid medium (Fig. 1D).

### Assessment of colony size

The colonies size in semisolid serum free media containing different concentrations of BMP-4 (0, 5, 10, 20 and 40 ng/ml) were 170.24 ± 70, 197.12 ± 73.92, 266.56 ± 58.80, 330 ± 96 and 258 ± 56.56 µm, respectively and there were significant differences between the control and BMP-4-containing groups. The largest colonies were observed in the group containing 20 ng/ml BMP-4 (*P*<0.05).

The colonies size in group with FBS and 0, 5, 10, 20 and 40 ng/ml BMP-4 were 97.6 ± 11.52, 140.76 ± 47.73, 172.80 ± 46.25, 155.90 ± 46.78 and 149 ± 43.58 µm respectively and satatistically differences were seen in the control and BMP-4-containing groups (*P*<0.05).

### Assessment of total number of colony

**Figure 2:**
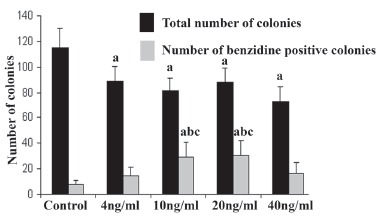
Total number and benzidine positive colonies formed after differentiation of EBs cells in serum free medium with different concentrations of BMP-4. Data are given as mean ± SD. a, Significant difference with control in the same column (*P*<0.05); b, Significant difference with 5 ng/ml group in the same column (*P*<0.05); c, significant difference with 40ng/ml group in the same column (*P*<0.05).

**Figure 3:**
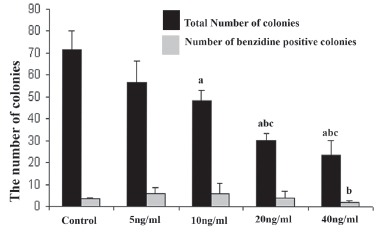
Total number and benzidine positive colonies formed after differentiation of EBs cells in semisolid media supplemented with FBS and different concentrations of BMP-4. Data are given as mean ± SD; a, Significant difference with control in the same column (*P*<0.05); b, Significant difference with 5ng/ml group in the same column (*P*<0.05); c, significant difference with 10ng/ml group in the same column (*P*<0.05).

The number of colonies which formed in serum free media with 0, 5, 10, 20 and 40 ng/ml BMP-4 were 115.13 ± 14.89, 88.75 ± 11.90, 81.5 ± 9.48, 87.75 ± 11.32 and 72.75 ± 11.48 respectively (Fig. 2) and these number in media supplemented with FBS and previously concentrations of BMP-4 were 71.60 ± 8.53, 56.80 ± 9.44, 48.40 ± 4.60, 30.40 ± 2.80 and 23.40 ± 7.1 respectively (Fig. 3). There were significant differences between the total number of colonies in the control and BMP-4-containing groups in both serum free and serum supplemented groups (*P*<0.05).

### Assessment of benzidine-positive colonies

The number and percent of benzidine-positive colonies in serum free condition and supplementation with 0, 5, 10, 20 and 40 ng/ml BMP-4 were 7.38 ± 3.37 (6.41%), 14.50 ± 6.90 (16.33%), 29.25 ± 11.63 (35.88%), 30.75 ± 11.53 (35.04%) and 16.75 ± 8.27 (23.02%) (Fig. 2) and in FBS supplemented groups were 3.60 ± 0.54 (5%), 5.80 ± 2.46 (11%), 6 ± 4.60 (11.40%), 4.2 ± 2.80 (13.40%) and 1.70 ± 1 (7%) (Fig. 3) respectively. Significant differences were observed in serum free media supplemented with 10 and 20 ng/ml of BMP-4 than among other BMP-4 concentrations and its control groups (*P*<0.05). These rates were statistically different in serum supplemented media with 40ng/ml of BMP-4 and other concentration of BMP-4 and its control groups (*P*<0.05).

### Ultrastructure of colonies

**Figure 4:**
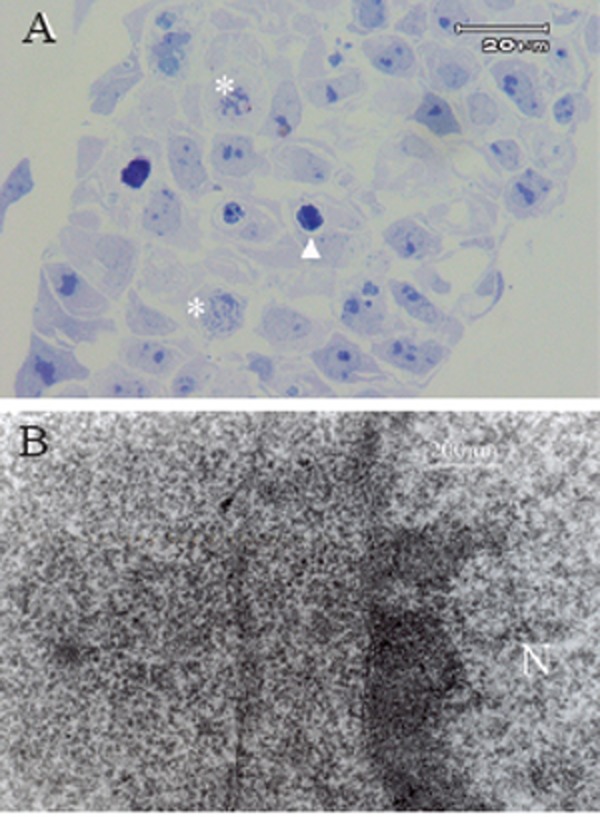
A, The light microscopic photograph of semi-thin section of a hematopoietic colony derived from ESCs treated with BMP-4 in serum free condition. Arrow shows the heterochromatin nucleus and its extrution from the cells and astretics showed the mitotic figures in this colony; B, Electron micrograph of proerythroblast cells differentiated from BMP-4-treated ESCs, showing the euchromatin nucleous and hemoglobin in its cytoplasm (less dense background of hemoglobin; N: nucleus).

Mitotic figures were frequently seen in semi-thin sections of hematopoietic colonies and the extrude of nucleus were seen in some cells within colonies (Fig. 4A). The electron microscopic features of the colony cells were those of erythroid like-cells. Their nuclei were round and euchromatic with a large nucleolus. The main constituents of the cytoplasm were polyribosomes and hemoglobin, whereas the relative content of other organelles, such as rough endoplasmic reticulum and mitochondria, was lower (Fig. 4B).

### One step real time PCR

Following gel electrophoresis, we found that all erythropoiesis related genes (epsilon and beta H1 globin, Runx1, beta major globin genes) and housekeeping gene (β2m) were expressed in the colonies in serum free and serum supplemented media but in the EBs cells which were not treated with BMP-4 (the control group) the βmajor globin gene was not expressed.

**Figure 5:**
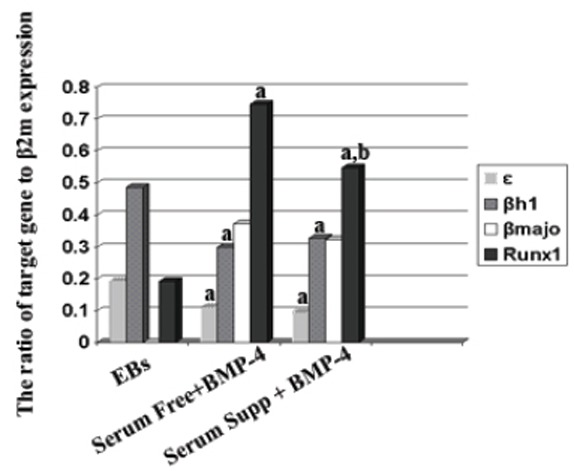
The ratio of genes expression erythropoiesis related genes (epsilon and beta H1 globin, Runx1, beta major globin genes) to β2m were shown in three groups of study (EBs; embryoid bodies cells (control), serum free media with BMP-4 and serum supplemented media with BMP-4). a, Significant differences with EBs or control group (*P*<0.05); b, Significant differences with serum free group (*P*<0.05).

After obtaining the standard curve and determination of Cycle Thresholds (C_t_) for each gene, relative quantitation of one step real time PCR were done using Pfaffl method and the ratio of target genes to β2m were calculated and shown in Fig. 5.

A high ratio of ε-globin to β2m gene expression were observed in embryoid bodies cells (0.191 ± 0.045) and there were significant differences between control (EBs cells) and the serum free (0.110 ± 0.032) and serum supplemented media (0.093 ± 0.022; *P*<0.05).

As were shown in Fig. 5 the ratio of βH1 to β2m gene expression in embryoid bodies cells was 0.485 ± 0.071 and there were significant differences between EBs cells with the colonies were formed in serum free (0.299 ± 0.052) and serum supplemented media (0.327 ± 0.028) in these regards (*P*<0.05).

The ratio of Runx1 to β2m gene expression was significantly increased in both BMP-4 treated groups: in serum free (0.747 ± 0.142) and serum supplemented groups (0.548 ± 0.079) in comparison to embryoid body's cells (0.192 ± 0.144; *P*<0.05). However this ratio was higher in serum free group than serum supplemented group (*P*<0.05).

The βmajor globin gene was expressed in both serum free (0.372 ± 0.047) and serum supplemented groups (0.321 ± 0.029) but there was no expression in embryoid body's cells. No significant differences were observed between the ratio of this gene expression in serum free and serum supplemented group.

## DISCUSSION

The potential of human, nonhuman primate and murine embryonic stem cells to differentiate to hematopoietic cells were widely investigated using different culture conditions (5, 6, 15, 20). In this study, we evaluated and compared the effects of different dosage of BMP-4 on mouse ESC induction to erythroid like cells in serum free and serum supplemented media.

Our results showed that the larger size of colonies were observed in the presence of 10 and 20 ng/ml BMP-4 in serum supplemented and serum free conditions, but in both conditions the total number of colonies was lower than their control. These observations suggested that differentiation of ESCs in the presence of BMP-4 caused an increase in the size of colonies especially in the serum free condition but it did not affect on the number of the colonies. On the other words the size of colonies which were formed in serum free condition was almost 1.5–2 folds larger than the control. It means that the cell population of the colonies in serum free condition was increased during culture period and this is due to the effect of BMP-4 as a proliferative factor and this effect was prominent in serum free condition. Similar results were reported by Chadwick *et al*. (12), they showed that the treatment of hESCs with BMP-4 during embryoid bodies development enhanced in the progenitor self renewal.

Our results showed that the percent of erythroid-like colonies in both BMP-4 treated groups was higher than their controls, indicating that the BMP-4 alone enhances the differentiation of erythroid like cells; however in the serum supplemented group may be due to presence of some inhibitory factor the differentiation of ESC to erythroid lineage was lower than serum free condition. Nakayama *et al*. (27) showed although BMP-4 is a principle hematopoietic regulator however its effect on the hematopoietic differentiation was observed when the culture media of mouse ESC was free of animal serum.

Our data for the first time showed the ability of BMP-4 alone for erythroid differentiation induction of ESC whereas the previous investigations had shown that BMP-4 with combination of other cytokines was required for hematopoietic differentiation (11, 12, 19, 20).

Chadwick *et al*. (12) reported that the addition of BMP-4 to the culture medium of human ESCs during EBs development had no statistically significant effect on hematopoietic differentiation but the treatment of human ESCs with a combination of cytokines and BMP-4 strongly induced hematopoietic differentiation.

Real time PCR was performed to estimate the level of gene expression in differentiated erythroid–like cells in both serum free and serum supplemented and control (EBs) groups and this technique provided us additional evidence in this study.

Our results showed that, all erythropoiesis related genes including epsilon and β H1 globin, Runx1, βmajor globin genes were expressed in the colonies of both systems. Since the ratio of Runx1 to β2m gene expression was higher in serum free group than serum supplemented group (*P*<0.05) and the other genes expression ratios did not show any significant difference between these two groups. Runx1 is an important regulator of hematopoietic development and plays a critical role in the development of both primitive and definitive hematopoietic cells.

This finding indicates that two pattern of erythropoiesis (primitive and definitive) were seen in serum free and serum supplemented conditions on the other hands these results suggested that the cells in both systems exhibit similar gene expression patterns. By contrast Kaufman *et al*. (28) showed that primitive erythroid colonies were not formed in serum free condition but definitive erythroid colonies were generated under this condition from primate ES cells. This difference may be due to different culture systems or different ES cell lines which have been used in these investigations. Adelman *et al*. (22) indicated that erythroid-specific markers such as βH1 and βmajor are expressed in serum-substituted conditions and hemoglobinization is reduced in the absence of serum. Also they concluded that the expression of βmajor globin was occurred only in the combination of BMP-4 with VEGF and SCF. They also showed that in the presence of FBS the level of BMP-4 expression increased endogenously in EBs cells (10-fold) and this level was sufficient to induced ES cells differentiation.

Also it was shown that the gene expression profile during ES cells differentiation to hematopoietic is a dynamic process (29–31). Thus ES cells during their differentiation in response to different levels of BMP-4 change their gene expression profile. Li *et al*. (20) showed BMP-4 stimulated expression of different hematopoiesis related genes among the differentiation of rhesus monkey ES cells colonies.

Our results showed that the primitive erythropoiesis related genes were expressed in embryoid bodies but the βmajor globin gene was not expressed. It may be culturing of the EB cells in the medium without LIF, differentiation occurred de novo especially primitive erythropoiesis.

These findings in combination with other part of our results (benzidine staining) showed that in serum free condition the effect of BMP-4 on the erythroid differentiation was prominent in comparison with serum supplemented group.
